# Immunotherapy Combined With Chemotherapy for Postoperative Recurrent Penile Squamous Cell Carcinoma: A Case Report and Literature Review

**DOI:** 10.3389/fonc.2022.837547

**Published:** 2022-03-23

**Authors:** Na Li, Tangpeng Xu, Zhen Zhou, Ping Li, Guohua Jia, Xiangpan Li

**Affiliations:** ^1^ Cancer Center, Renmin Hospital of Wuhan University, Wuhan, China; ^2^ Department of Cardiology, Renmin Hospital of Wuhan University, Wuhan, China; ^3^ Department of Oncology, Renmin Hospital of Wuhan University, Wuhan, China

**Keywords:** penile squamous cell carcinoma, lymph node metastasis, programmed death ligand-1, immunotherapy, chemotherapy

## Abstract

Penile squamous cell carcinoma (SCC) is a rare malignant tumor in males with a poor prognosis. Currently, the primary treatment is surgery. Recurrent cases have limited treatment options after failed radiotherapy and chemotherapy. The therapeutic effect of immunotherapy in penile SCCs has not been reported. Tislelizumab, a new PD1 inhibitor, has shown a satisfactory impact in treating head and neck SCC and lung SCC combined with chemotherapy. However, there is currently no report on its efficacy in penile SCC. Here, a 76-year-old man with multiple enlarged inguinal lymph nodes 11 months after radical surgery for penile SCC was administered immunotherapy (tislelizumab) combined with chemotherapy (albumin paclitaxel plus nedaplatin) for 2 cycles. Pelvic Magnetic resonance imaging (MRI) showed that the multiple lymph nodes in the groin area disappeared. To our knowledge, this is the first case report of immunotherapy combined with chemotherapy showing promising results in recurrent penile SCC. It provides a basis for developing a new treatment option combining immunotherapy and chemotherapy, whose efficacy needs to be further evaluated in penile SCC.

## Introduction

Penile cancer is a rare malignant tumor with a global incidence of 1-10/100000 (1). Penile SCC is the most common pathological type of penile malignancies, accounting for about 95% of all penile cancer cases (2). The prognosis of patients with penile SCC is closely related to metastasis to inguinal lymph nodes. There are 4-25 superficial inguinal lymph nodes receiving drainage from preputial and penile shaft skins (3). Most patients with positive inguinal lymph nodes have inguinal lymph node recurrence within a short period after radical surgery. Such patients often have a poor prognosis.

Rescue treatment methods include chemotherapy, radiotherapy, surgery, targeted therapy, or combinations (4–6). Tislelizumab, the first PD-1 monoclonal antibody that successfully modifies the Fc segment, has a high affinity for PD-1. Tislelizumab monotherapy for advanced solid tumors shows acceptable safety and tolerance (7). It has been reported that immunotherapy combined with chemotherapy has a significant effect on advanced head and neck SCC (8). However, there is no report on its therapeutic effect in penile SCC. A patient with inguinal lymph node metastasis 11 months after radical resection of penile SCC was admitted to our hospital in June 2021. After immunotherapy combined with chemotherapy, he achieved complete remission (CR) with no severe adverse drug reactions. Based on previous literature, we report this case and discuss the progress in treating advanced penile SCC.

## Case Presentation

**Figure 1:**
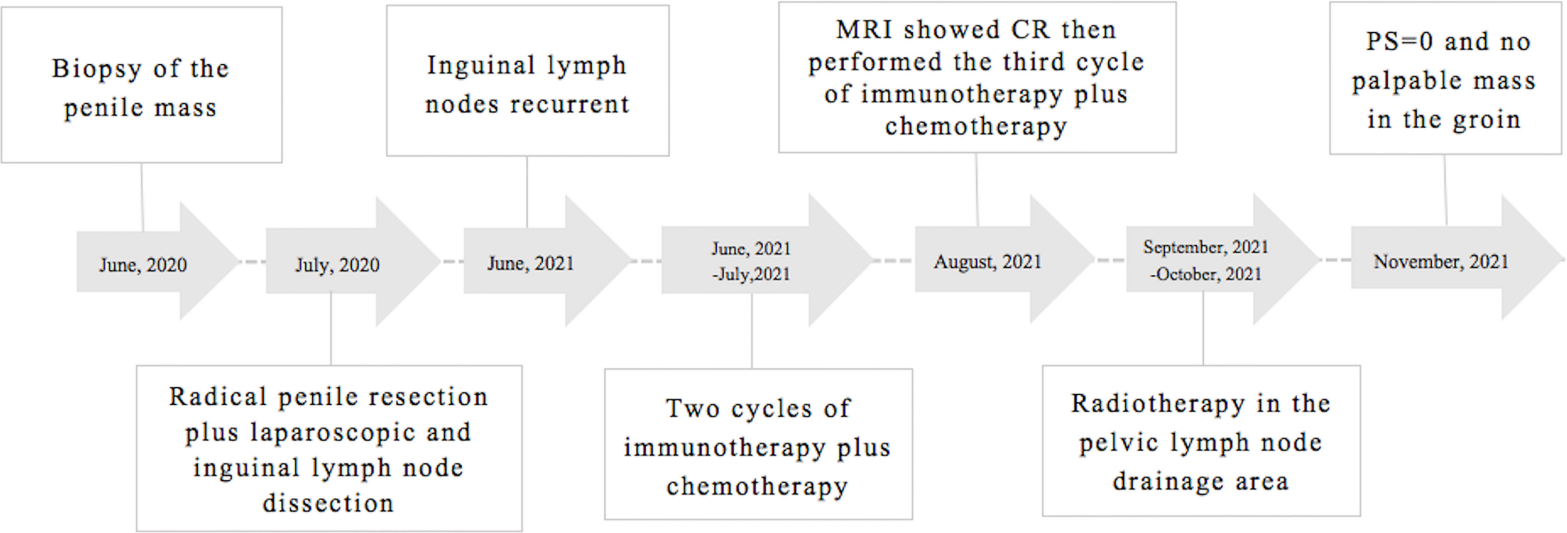
Treatment timeline of the case.

**Figure 2:**
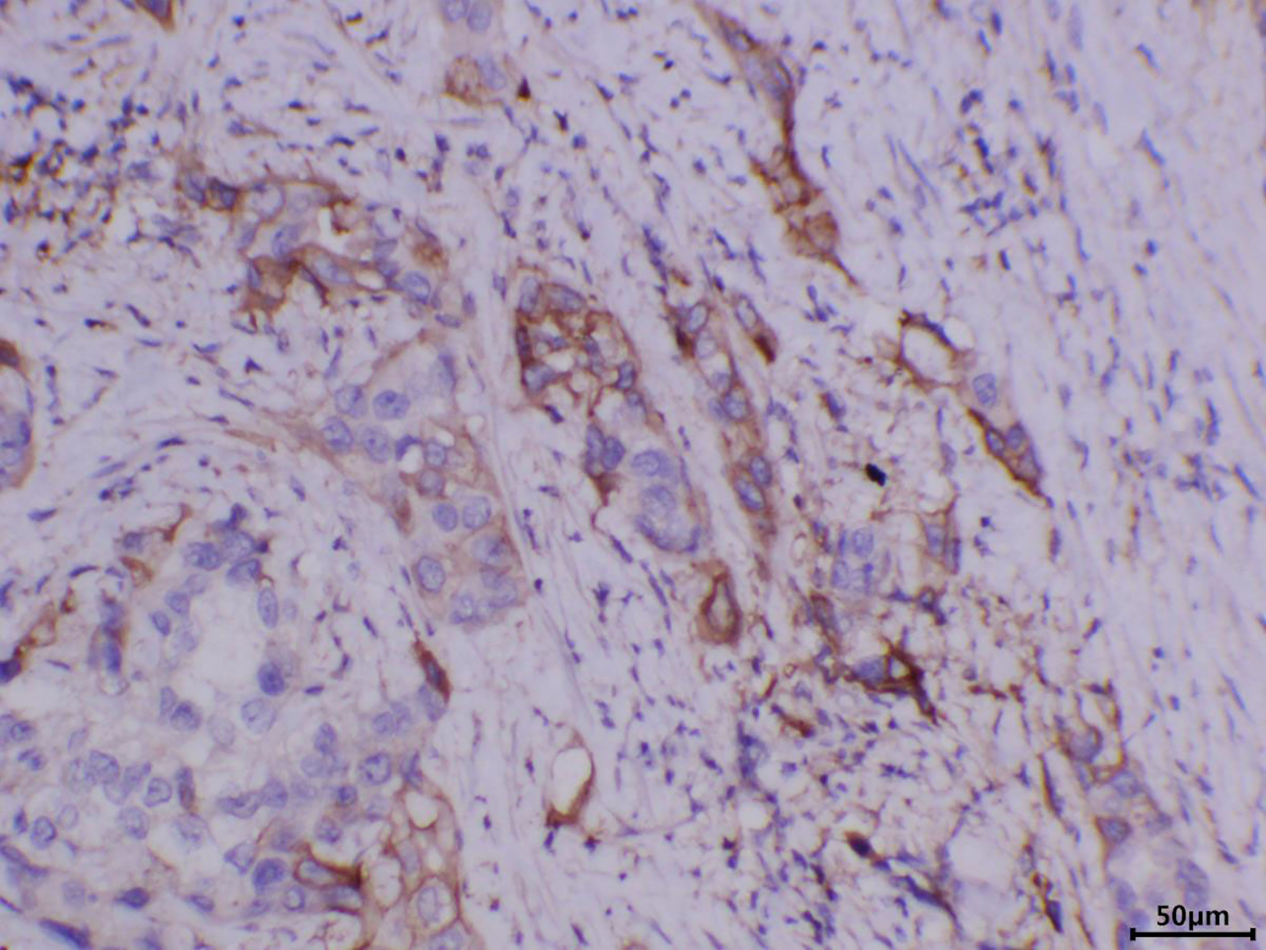
Immunohistochemistry showing that PD-L1 was expressed in the primary penile squamous cell carcinoma tissue, TPS=10%.

**Figure 3:**
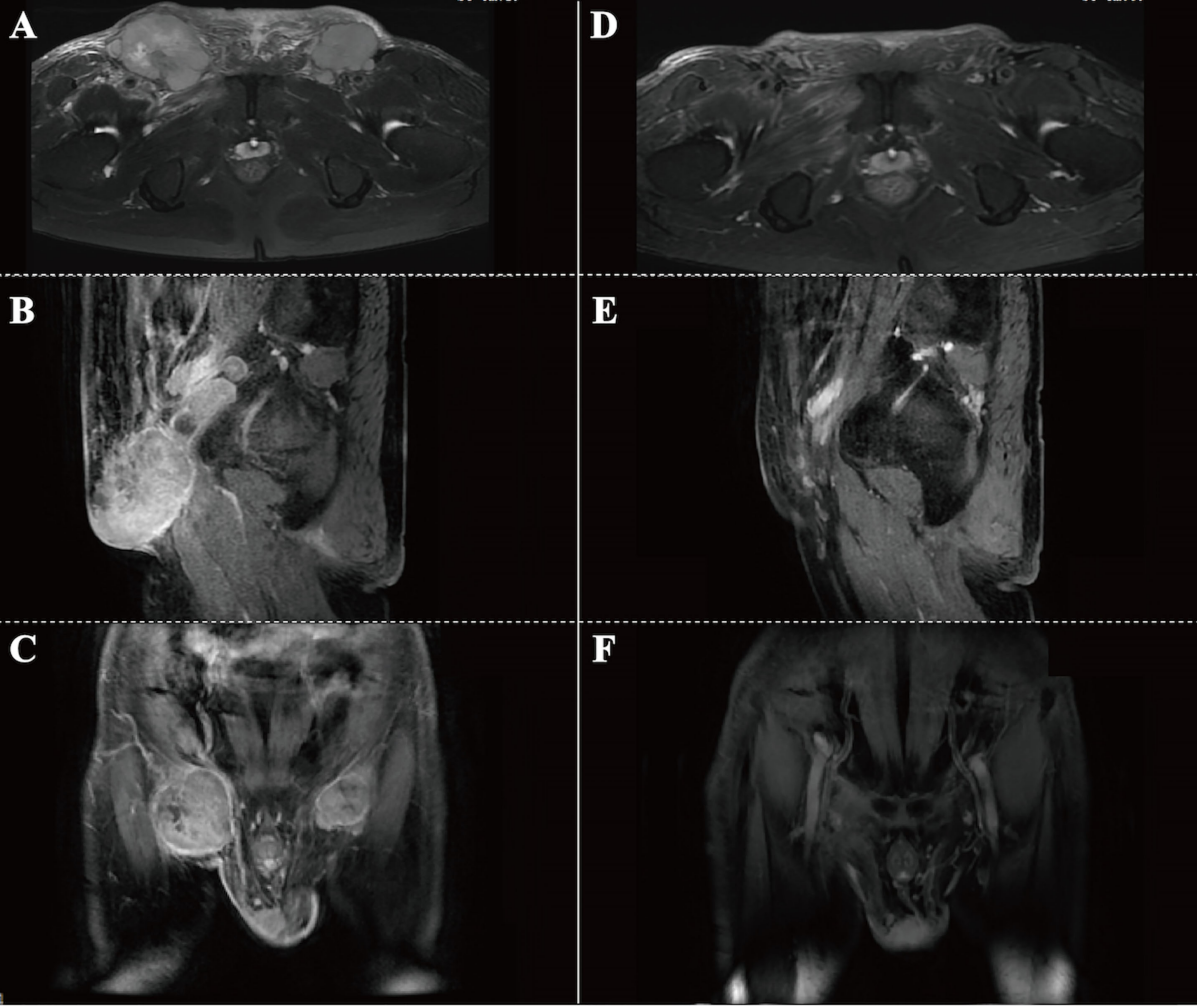
Pelvic MRI results. **(A-C)** MRI before treatment on June 12, 2021. Horizontal **(A)**, sagittal **(B)** and coronal **(C)** images showing multiple enlarged lymph nodes in the groin area on both sides. **(D-F)** MRI after 2-cycle treatment on August 7, 2021. Horizontal **(D)**, sagittal **(E)** and coronal **(F)** images showing the disappearance of swollen inguinal lymph nodes in the groin.

**Figure 4:**
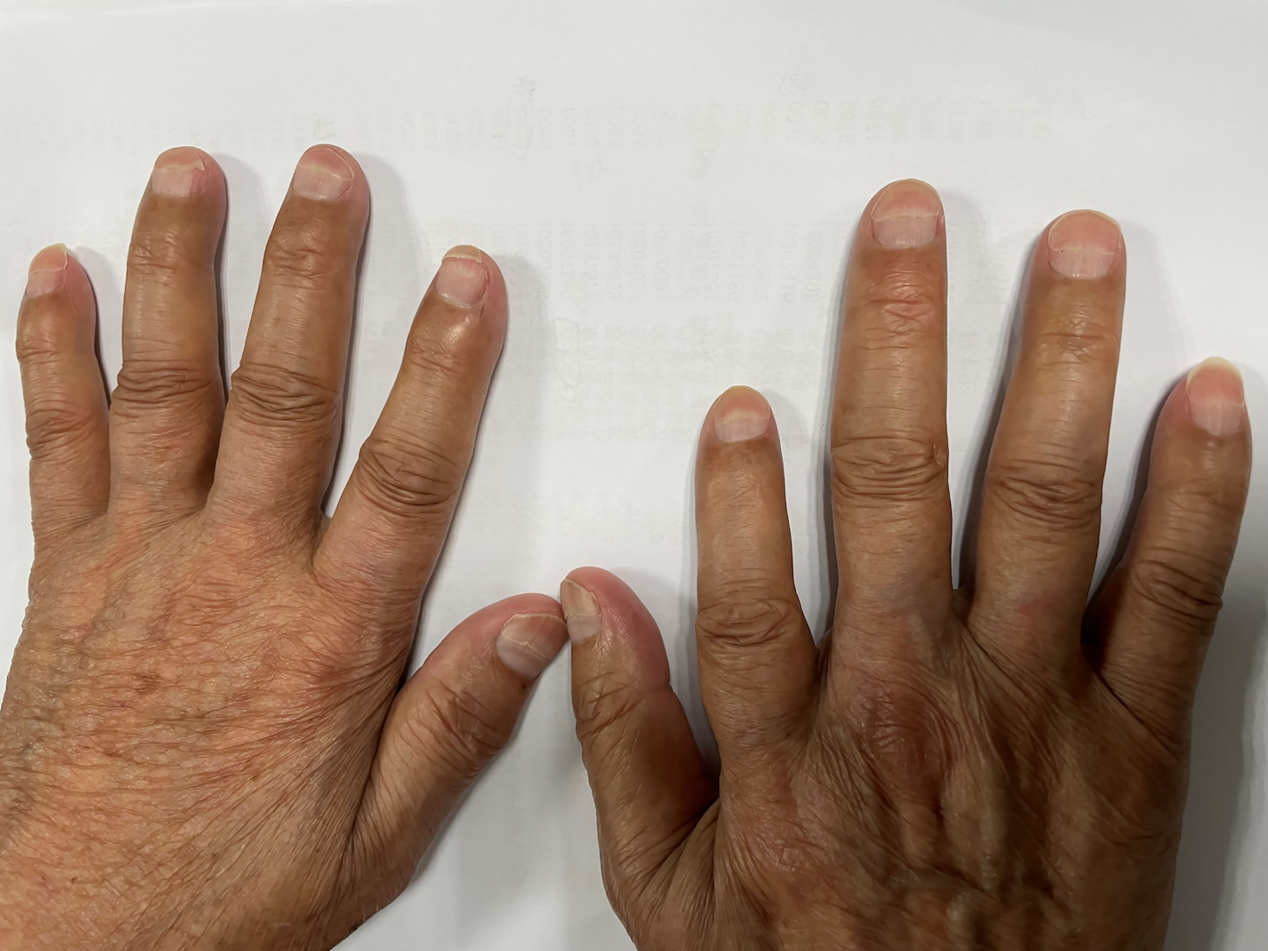
Broken nails on the patient’s hands after combined treatment.

A 76-year-old male patient was admitted to the People’s Hospital of Yangxin County, Hubei province, for “penile mass with progressive dysuria for one month” in June 2020. The treatment history of this patient is shown in **Figure 1**. Transurethral resection of the prostate and biopsy of the penile mass was performed. Pathological examination revealed poorly differentiated SCC with massive necrosis. Then, the patient was treated at Union Hospital of Tongji Medical College, Huazhong University of Science and Technology. In July 2020, he underwent radical penile resection plus laparoscopic lymph node dissection plus inguinal lymph node dissection under general anesthesia. Immunohistochemical staining showed cancer cells were P40(+) and CK5/6(+). Postoperative pathological examination showed poorly differentiated SCC of the penis (**Supplementary Figure 1**). The cancer tissue mainly invaded the lamina propria and focally invaded the cavernous body of the urethra. There was no invasion on the cut edges of the urethra, corpus cavernosum, and penile skin. One-third of the left and right inguinal lymph nodes showed cancer metastasis. The tumor stage was pT_2_N_2_M_0_, IIIB (AJCC eighth edition TNM staging) (9). The patient refused adjuvant chemotherapy because he had an advanced age. In June 2021, a follow-up examination revealed enlarged inguinal lymph nodes, and the patient was admitted to Renmin Hospital of Wuhan University. Programmed death ligand-1 (PD-L1) staining of the previously resected tumor specimens showed low levels in the primary penile SCC tissue (TPS=10%) (**Figure 2**). On June 12, 2021, pelvic MRI results showed multiple lymph nodes adjacent to bilateral iliac vessels and groin areas (**Figures 3A–C**). After obtaining the patient’s informed consent, the first cycle of immunotherapy plus chemotherapy (tislelizumab 200 mg d1; albumin paclitaxel 400 mg d1 plus nedaplatin 120 mg d1-2) was started on June 16, 2021. The patient developed grade 2 myelosuppression, grade 3 general fatigue, and grade 2 lethargy after the first cycle of chemotherapy, mainly due to an advanced age. CA125 levels on July 9, 2021, were 40.9 U/ml. The second cycle of dose-reduced immunotherapy plus chemotherapy (tislelizumab 200 mg d1; albumin paclitaxel 200 mg d1 plus nedaplatin 100 mg d1-2) was started on July 15, 2021. On August 6, 2021, CA125 levels were 11.4 U/ml, i.e., lower than the previous value. Pelvic MRI results after the second cycle of treatment on August 7, 2021, showed that previously detected multiple lymph nodes adjacent to bilateral iliac vessels and in the groin area were not detectable (**Figures 3D–F**). Treatment effectiveness was evaluated as CR. On August 9, 2021, the third cycle of immunotherapy plus chemotherapy (tislelizumab 200 mg d1; albumin paclitaxel 200 mg d1 plus nedaplatin 100 mg d1-2) was initiated. Radiotherapy in the pelvic lymph node drainage area (CTV: 50GY/25F/2GY) was performed from September to October 2021. In September 2021, the patient had broken nails on both hands (**Figure 4**), which was tolerable and improved one week later. Radiotherapy ended in October 2021, and treatment effectiveness was evaluated as CR. There were no immunotherapy-related side effects. The patient had grade 1 radiodermatitis. In November 2021, the patient was followed up by phone, and his PS score was 0, with no palpable mass in the groin.

## Discussion

The postoperative staging of this penile SCC case was pT_2_N_2_M_0_, stage IIIB. The patient refused standard postoperative chemotherapy and radiotherapy. The disease relapsed one year later. There was a massive fusion of lymph nodes in the double groin, accompanied by noticeable swelling and pain, as well as local ulcers. The patient declined further biopsy. According to the NCCN Clinical Practice Guidelines in Oncology for Penile Cancer, Version 1.2021, the paclitaxel, ifosfamide, and cisplatin (TIP) combination is the preferred option for postoperative chemotherapy in penile cancer patients with positive inguinal lymph nodes. However paclitaxel and cisplatin/carboplatin administration was adequate and had less toxic side effects in patients with high-risk penile tumors (10). Studies have shown that patients with SCC can benefit from tislelizumab regardless of PD-L1 expression (11). The patient underwent 3 cycles of albumin paclitaxel plus nedaplatin combined with immunotherapy and achieved clinical CR. However, due to advanced age, general fatigue, and lethargy, the patient could not tolerate complete and sufficient cycles of chemotherapy; therefore, chemotherapy was terminated, and local radiotherapy was performed. It was well-tolerated and achieved sound effects.

Early penile SCC is mainly treated by surgery, and the overall 5-year survival rate can reach 90%, while the 5-year survival rate of patients with positive lymph nodes is only about 30% (12). Surgical treatment is the cornerstone of treating primary tumors and lymph node metastases in patients with penile SCC. The multimodal treatment of neoadjuvant chemotherapy and adjuvant chemoradiotherapy plays an increasingly important role in treating penile SCC (1). The postoperative recurrence rate in penile SCC patients without lymph node metastasis is 2.3%; meanwhile, in patients with lymph node metastasis is 19.1%, with a 5-year survival rate of 32.7%, and median survival of patients with distant recurrence is 22 months (13). The surgical method for treating penile SCC is determined by the primary tumor’s size, location, pathology, and stage. In stage Ta/T1a cases, it is recommended to perform resection of the local lesion while preserving the penis. Maintaining the penis may increase the local recurrence rate, but it hardly affects long-term survival (14). In stage T_1_/T_2_ patients without lymph node metastasis and local lesions < 4 cm, radical radiotherapy can achieve a 70-90% local control rate. Rescue surgery is feasible for patients with local recurrence, and radiotherapy can be used as an alternative to penis preservation surgery (15). In patients with positive lymph nodes, inguinal lymph node dissection is strongly recommended, and chemotherapy should be performed after surgery in patients with positive pathological lymph nodes. Patients who cannot undergo resection surgery or show disease progression, systemic chemotherapy, local radiotherapy, or participation in clinical trials are recommended (16). A retrospective study involving 19 cases showed that compared with palliative radiotherapy, docetaxel plus cisplatin plus cyclophosphamide as a neoadjuvant chemotherapy regimen allows patients to receive penile resection surgery after partial remission, which significantly prolongs progression-free survival (PFS) and overall survival (OS) (17). A phase 2 clinical trial involving 30 patients with pN_2_/N_3_ penile SCC used paclitaxel, ifosfamide, and cisplatin as neoadjuvant chemotherapy, and 50% of patients achieved objective remission, 10% of whom achieved CR, and time to progression (TTP) and OS in remission cases were significantly improved (18). A case report further showed the effect of neoadjuvant chemotherapy. A Japanese patient with T_3_N_3_M_0_ penile SCC not suitable for radical resection of inguinal metastasis was administered paclitaxel, ifosfamide, and cisplatin as neoadjuvant chemotherapy, and the lesion was significantly reduced. The patient then underwent bilateral and pelvic lymph node dissection and was still alive after 8 months with no signs of recurrence (19).

A previous study showed that platinum plus paclitaxel as adjuvant chemotherapy was safe and effective in patients with high-risk penile tumors, including tumor expansion, bilateral lymph node metastasis, pelvic lymph node metastasis, and R1 resection. The 2-year overall survival rates of inguinal lymph node positive patients administered platinum plus paclitaxel as adjuvant chemotherapy and surgery alone were 46% and 28%, respectively. Compared with other adjuvant chemotherapy regimens, paclitaxel and cisplatin/carboplatin have less toxic side effects (10). A retrospective study of 140 cases of penile SCC confirmed the high status of platinum in chemotherapeutic regimens for penile SCC. Patients receiving cisplatin-based chemotherapy have more prolonged overall survival and better prognosis (20). In a previous study, the median OS of patients with positive pelvic lymph nodes administered postoperative adjuvant chemotherapy was 21.7 months. In comparison, the median OS of counterparts without chemotherapy was 10.1 months, indicating that postoperative chemotherapy can significantly improve patient prognosis (21). In another study, 11 patients underwent concurrent chemoradiotherapy after surgery, while 12 did not receive adjuvant therapy after surgery. Although there was no significant difference in OS between the two groups, 1-year and 2-year tumor-specific survival rates in patients receiving concurrent chemoradiotherapy (72.7% and 54.5%, respectively) were significantly higher than those of patients who did not receive adjuvant therapy (57.1% and 28.4%, respectively), indicating that postoperative concurrent chemoradiotherapy can increase local control rate and reduce distant metastasis (22).

After failed first-line treatment in patients with advanced penile SCC, the survival rate after salvage surgery or radiotherapy and chemotherapy is still meager. The median survival time is less than 6 months (23). Although adjuvant radiotherapy cannot replace adjuvant chemotherapy in patients with positive lymph nodes after radical resection (24, 25), radiotherapy can improve the regional control rate of pelvic lymph nodes, and palliative radiotherapy can improve local symptoms in patients (26–28). Advanced penile cancer cases with positive epidermal growth factor (EGFR) expression can benefit from EGFR-targeted drugs combined with chemotherapy. Cetuximab has anti-tumor effects in metastatic penile cancer and can enhance the clinical outcome of platinum-based chemotherapy (29, 30). During the treatment of a stage pT_2_N_2_ patient with inguinal recurrence after surgery, targeted therapy combined with chemotherapy (cetuximab 800 mg d1; paclitaxel 210 mg d1 plus cisplatin 40 mg d1-d3) significantly reduced inguinal masses. After another recurrence, chemotherapy alone showed no effects, but the tumor decreased again after combining targeted treatments (31).

In recent years, immunotherapy has shown encouraging effects in treating a variety of malignant tumors, especially in lung cancer and head and neck SCC. The overall survival of patients administered therapy is significantly prolonged (32, 33). The efficacy of immunosuppressive agents is usually related to high tumor mutation load, immune cell infiltration, and PD-L1 expression (34). As many as 40% of patients with penile SCCs are HPV positive (35). The integration of HPV leads to increased expression of oncogenes, and the high tumor mutation load caused by HPV can enhance the immune system’s recognition of tumors. A retrospective study of 213 penile SCC cases described the complexity of the tumor immune microenvironment in penile SCC. HPV occurrence in penile SCC is negatively correlated with PD-L1 expression, a significant predictor of lymph node metastasis; patients with high PD-L1 expression have a poor prognosis (36). A multi-center retrospective study involving 116 patients showed endogenous and exogenous PD-L1 expression in penile SCC tissues, and the positive rate of PD-L1 expression in penile SCC tissue was 53.4%. The 3-year tumor-specific survival rates of patients with and without PD-L1 expression were 50.6% and 77.4%, respectively, suggesting that the PD1/PD-L1 axis may be a potential therapeutic target for penile SCC (37).

At present, there are few studies applying immune checkpoint inhibitors (ICIs) in penile SCC. Trafalis et al. reported the efficacy of an immune checkpoint inhibitor in a patient with penile SCC with failed comprehensive treatment after recurrence. After surgery, adjuvant chemotherapy, and complementary radiotherapy, the disease progressed, and nivolumab was administered at a starting dose of 3 mg/kg in a 14-day cycle. After 8 cycles of treatment, a good partial response rate of the tumor was obtained, and tumor volume was reduced by 80%. Finally, the patient died of septic shock. During immunotherapy, the patient tolerated the side effects well (38). A phase 1 clinical trial showed that cabozantinib combined with nivolumab (CaboNivo) and CaboNivo plus ipilimumab (CaboNivoIpi) is significantly more effective than single ICIs or cabozantinib in the treatment of metastatic urothelial carcinoma, with manageable overlapping toxicity at reasonable doses (39). However, in a multicenter phase 2 clinical trial, 5 patients with penile cancer had no treatment effects after combined treatment with nivolumab and ipilimumab (40). A Phase 2 clinical trial (NCT03866382) investigating the efficacy of cabozantinib and nivolumab combined with ipilimumab in metastatic rare genitourinary tumors is expected to be completed in 2023. There are currently several ongoing phase 2 clinical trials (NCT02721732, NCT03074513, and NCT03391479) evaluating the efficacy of ICIs in advanced penile SCC. In addition, a single-center phase 2 clinical trial (NCT03686332) involving 32 patients with advanced penile cancer is evaluating the efficacy of atezolizumab with or without radiotherapy in patients with unresectable advanced penile cancer. However, no relevant reports on immunotherapy combined with chemotherapy in advanced penile SCC are available. Immune cell dysfunction in the head and neck SCC tumor microenvironment can lead to immunosuppression (41).

The interaction of PD-L1 on tumor cells and PD-1 on cytotoxic T cells can block the activity of PI3K and Akt, disrupt glucose metabolism, and inhibit the production of Th1 cytokines, leading to dysfunction and exhaustion of effector T cells and protecting tumor cells from killing by CD8+ T cells (42). The primary function of immune checkpoint inhibitors is to enhance the toxic effect of CD8+T cells and reactivate the immune system against tumor cells (43). Chemotherapeutic drugs regulate the immune response of tumors in many ways, including the recruitment of dendritic cells (DC) to the tumor, the induction of tumor cell death, the release of tumor antigens, the induction of HMGB1 release in tumor cells, and the ectopic calreticulin; promote antigen uptake by DC and subsequent T cell stimulation, and deplete immunosuppressive cells and promote the transformation of immunosuppressive cell phenotypes (44). In addition to exerting immunomodulatory effects, doxorubicin, cisplatin, and paclitaxel can directly sensitize tumor cells to CTL attack and synergistic effects with cytotoxic T lymphoid associated protein 4 (CTLA-4) blockers to enhance the treatment effect of immunosuppressants (45). The immunostimulatory effects of chemotherapy and radiotherapy can improve the efficacy of many immunotherapies, including ICIs (46).

The KEYNOTE-048 study showed that the 4-year survival rate after treatment with immunotherapy plus chemotherapy was 4.3 times that of the EXTREME regimen in the treatment of head and neck SCC, and the efficacy of immunotherapy combined with chemotherapy was not related to PD-L1 expression. Immunotherapy combined with chemotherapy has broadened the clinical use of immunotherapy (8). The ESCORT-1st study showed that standard first-line chemotherapy for advanced or metastatic esophageal cancer had a median survival time of 12 months and a median PFS of 5.6 months; meanwhile, the median survival time after the addition of immune checkpoint inhibitor to the first-line chemotherapy regimen was 15.3 months, for a median PFS of 6.9 months, indicating that immunotherapy combined with chemotherapy could significantly prolong OS and PFS in these patients (47). In this case, after treatment with immunotherapy combined with chemotherapy, the patient achieved CR. It provides a novel strategy for the treatment of penile SCC.

## Conclusion

In this case, Tislelizumab combined with paclitaxel and platinum chemotherapy showed satisfactory effects in treating recurrent penile SCC. This is the first case report of immunotherapy combined with chemotherapy for treating advanced, recurrent penile SCC. The above results suggest that immunotherapy combined with chemotherapy has a good therapeutic effect in advanced, recurrent penile SCC. Still, further clinical trials are needed to evaluate the efficacy of immunotherapy combined with chemotherapy for advanced penile SCC.

## Data Availability Statement

The original contributions presented in the study are included in the article/**Supplementary Material**. Further inquiries can be directed to the corresponding author.

## Ethics Statement 

The studies involving human participants were reviewed and approved by the Human and Research Ethics Committees of the Renmin Hospital of Wuhan University. The patients/participants provided their written informed consent to participate in this study. Written informed consent was obtained from the individual(s) for the publication of any potentially identifiable images or data included in this article.

## Author Contributions 

NL collected data and drafted the manuscript. TX and ZZ provided figures and pathology results and drafted the manuscript. PL and GJ review the manuscript. XL designed the study and reviewed and critique the manuscript. All authors contributed to the article and approved the submitted version.

## Funding

This study was supported by the Natural Science Foundation of Hubei Province [No.2014CFB394, 2019CFB721], Health and Family Planning Commission of Hubei Province [No.WJ2017M027] and CSCO hausen Cancer Research Foundation [No.Y-HS202101-0079].

## Conflict of Interest

The authors declare that the research was conducted in the absence of any commercial or financial relationships that could be construed as a potential conflict of interest.

## Publisher’s Note

All claims expressed in this article are solely those of the authors and do not necessarily represent those of their affiliated organizations, or those of the publisher, the editors and the reviewers. Any product that may be evaluated in this article, or claim that may be made by its manufacturer, is not guaranteed or endorsed by the publisher.
