# Supplementary figures and images for: Immunotherapy Combined With Chemotherapy for Postoperative Recurrent Penile Squamous Cell Carcinoma: A Case Report and Literature Review
Source: Front Oncol. 2022 Mar 23;12:837547. doi: 10.3389/fonc.2022.837547 (PMC8984464; doi:10.3389/fonc.2022.837547)

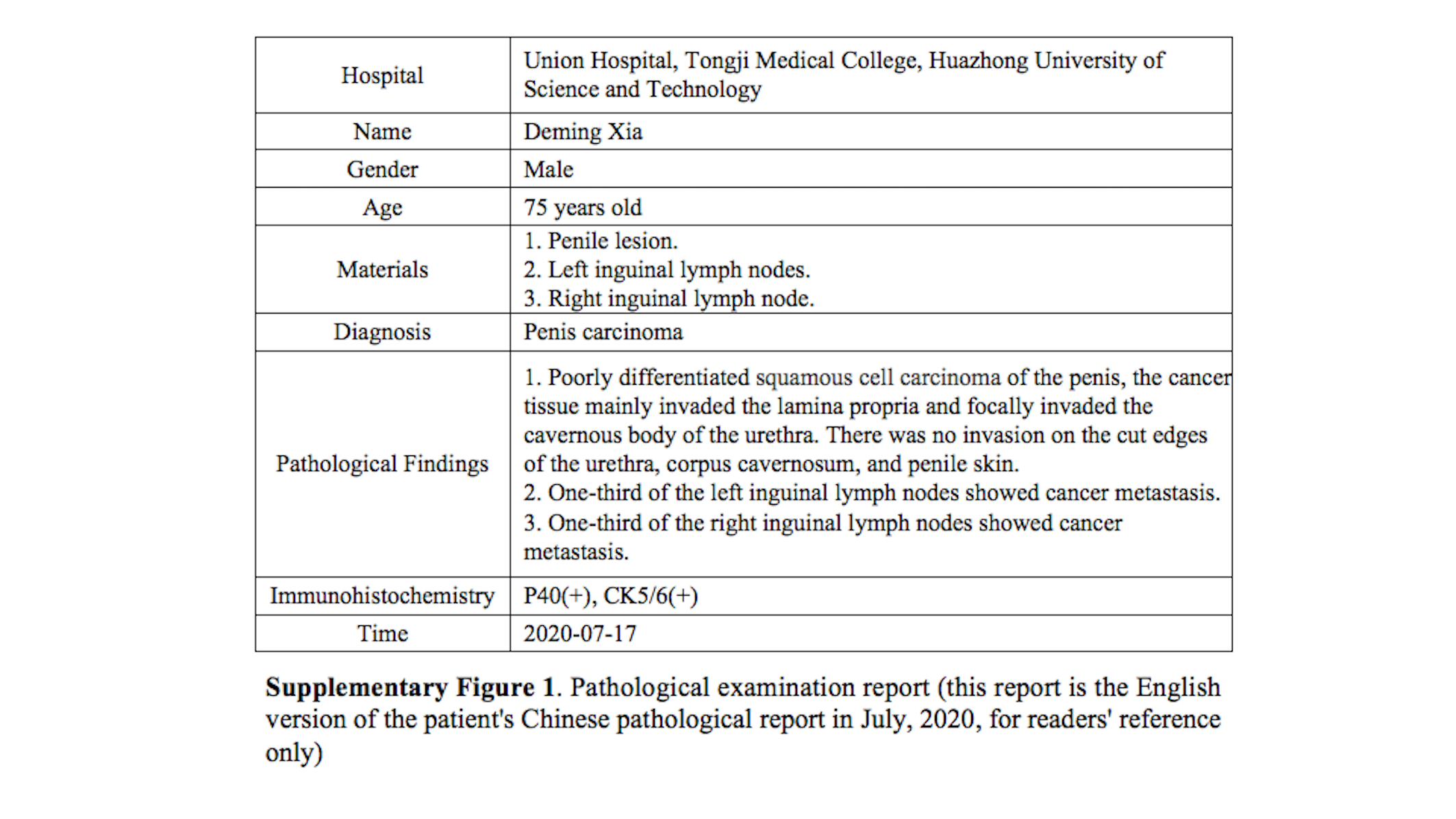

Supplement: Supplementary file 1 [file Image_1.tif]
